# Challenges to implementing planning processes in Brazilian health regions

**DOI:** 10.11606/s1518-8787.2023057005138

**Published:** 2024-04-01

**Authors:** Oswaldo Yoshimi Tanaka, Marco Akerman, Marília Cristina Prado Louvison, Aylene Bousquat, Nicanor Rodrigues da Silva Pinto, Ana Lígia Passos Meira, Lídia Pereira da Silva Godoi, Ana Paula Chancharulo e Morais Pereira, Sandra Maria Spedo, Monique Batista de Oliveira, Ilana Eshriqui, Marcio Anderson Cardozo Paresque

**Affiliations:** I Universidade de São Paulo Faculdade de Saúde Pública São Paulo SP Brasil Universidade de São Paulo. Faculdade de Saúde Pública. São Paulo, SP, Brasil; II Universidade Federal de São Paulo Programa de Pós-Graduação em Saúde da Família São Paulo SP Brasil Universidade Federal de São Paulo. Programa de Pós-Graduação em Saúde da Família. São Paulo, SP, Brasil; III Universidade do Estado da Bahia Departamento de Ciências da Vida Salvador BA Brasil Universidade do Estado da Bahia. Departamento de Ciências da Vida. Salvador, BA, Brasil; IV Hospital Israelita Albert Einstein Centro de Estudos Pesquisa e Prática em Atenção Primária à Saúde e Redes São Paulo SP Brasil Hospital Israelita Albert Einstein. Centro de Estudos Pesquisa e Prática em Atenção Primária à Saúde e Redes. São Paulo, SP, Brasil

**Keywords:** Primary Health Care, Regionalization Health Planning, Health Planning Support

## Abstract

**OBJECTIVE:**

To recognize elements that facilitated or hindered the PlanificaSUS implementation stages.

**METHODS:**

A multiple case study was carried out in four pre-selected health regions in Brazil—Belo Jardim (PE), Fronteira Oeste (RS), Sul-Mato-Grossense (MT) and Valença (BA) using systemic arterial hypertension and maternal and child care as tracer conditions. Participant observation (in regional interagency commissions) and in-depth interviews with key informants from state and municipal management and primary health care and specialized outpatient care service professionals within the project were carried out in these four regions. Analysis was built according to political, technical-operational, and contextual dimensions.

**RESULTS:**

The political dimension evinced that the regions found the project an opportunity to articulate states and municipalities and an important political bet to build networks and lines of care but that there remained much to be faced in the disputes related to building the Unified Health System (SUS). In the technical operational dimension, it is important to consider that primary health care stimulated a culture of local planning and favored traditional tools to organize and improve it, such as organizing registrations, agendas, and demands. However, centralized training and planning-inducing processes fail to always respond to local needs and can produce barriers to implementation.

**CONCLUSIONS:**

It is worth considering the central and regional role of state managers in the commitment related to the project and the effect of mobilizing primary health care and expanding its power. There remains much to be faced in the disputes at stake in bullring SUS.

## INTRODUCTION

Health systems, especially those based on the concept of health as a human right, face numerous and growing challenges. One of the biggest certainly refers to responding to populations’ illness profile due to demographic and epidemiological transitions. Notably, the trajectories of changes and adjustments in health systems occurred non-linearly, moving from innovative proposals to mere technical and managerial adjustments^1^.

In Brazil, the health status characterized by its triple burden of disease accelerated population aging, and care fragmentation also pose challenges for its Unified Health System (SUS). Thus, strategies to integrate care and change health work processes (especially to manage chronic conditions) are essential to ensure quality of care and improve a health system^2^ that aims at equality, universality, and comprehensiveness.

Articulating health services is essential to ensure care comprehensiveness and continuity^3^, especially integrating specialized outpatient care (SOC) and primary health care (PHC), which has been considered a neuralgic point of the system^4^. This integration goes beyond a simple administrative procedure as results differ between countries.

The Brazilian proposal for comprehensiveness to ensure continuity of care was based on the constitution of regionalized health care networks (HCN), conceiving PHC as the ordering and coordinating center of care offering SOC. However, this proposal faces insufficient supply, heterogeneous territorial distribution, and organization forms of SOC services^5^.

Brazilian and international^1,11^ experiences to integrate PHC and SOC have aimed to change this scenario. Thus, analyzing them is crucial to understand the complex construction processes of HCN. A Brazilian experience that stands out is its Health Care Planning (HCP), which the National Council of Health Secretaries proposed as a methodology to organize services and integrate HCN^14^. HCP actions, until then called “Planning of Primary Health Care in the States,” were initially directed to PHC and focused on management^16^. The design of HCN guidelines included SOC in this process^17^.

Of the HCP proposals implemented in Brazil, “PlanificaSUS,” a project of the Support Program for the Institutional Development of the Unified Health System that was developed by Hospital Israelita Albert Einstein in partnership with the National Council of Health Secretaries, stands out for its scope and number of actors.

Its first triennium (2018–2020) was carried out in 26 health regions in Brazil to implement HCP, strengthen PHC and its network organization with SOC^18^. Thus, this study aims to analyze the PHC-SOC integration implementation experience and find its hindering and facilitating elements^17,21^.

## METHODS

A multiple case study^22,23^was conducted from 2019 to 2020 in four of the 27 Brazilian health regions in the PlanificaSUS project (one region withdrew after being chosen). This choice was intentionally made by the researchers and corroborated by the project coordination. Considering the state of development and existence of a municipality chosen as the headquarters of the project in each state and the location of the units, laboratories, and services in which PlanificaSUS actions were being developed, a decision was made to continue studying these hub municipalities and choosing one more in the region, which only offered primary care services and depended on the SOC of the hub municipality.

In total, 56 indicators were initially collected from the Datasus and Region and Networks Research System databases: Path to Universalizing Health in Brazil^24^. These indicators included service supply; outpatient activities; hospital activities; live births; and mortality rate by cause.

Structure, process, and result data were collected and analyzed for coherence, trend, and relations between the activities of care complexities. These variables were analyzed according to the priority lines of care for maternal and child care (Rede Cegonha)^25^and that for hypertension and diabetes, which were chosen for their importance in the Brazilian epidemiological scenario^29^.

Based on the analysis of these indicators, 12 health regions were pre-selected for their contradictions between health service provision and morbidity and mortality indicators, which could contribute to show health care process characteristics and the contextual variables influencing the implementation of health actions and policies.

The final choice of the health regions was carried out in a workshop involving the team of researchers and the coordinating team of the PlanificaSUS project, who helped to guide the choices by a better evaluation of their political, operational, and contextual characteristics. In total, four health regions in four states (Bahia, Mato Grosso, Pernambuco, and Rio Grande do Sul) were selected in this process. Study cases were chosen from two regions that prioritized maternal and child health care (Mato Gross and Rio Grande do Sul) and two, care for people with hypertension and diabetes (Bahia and Pernambuco) according to PlanificaSUS.

An evaluation matrix was elaborated for each region and key informants were found for field interviews based on available information.

Research fieldwork was planned in remote meetings with interlocutors from State Health Secretariats (SHS) who acted as a technical reference for the PlanificaSUS project in the chosen federative units. The research design dialogued with the main characteristics and objectives of the project, which seeks to integrate and strengthen macro and micro PHC processes and integrate it in a network offering SOC. For this, specific HCP instruments, such as dashboards, which can quickly visualize indicators to monitor teams’ daily performance of the teams^13^, were used. Process management methodologies, such as the PDSA (plan-do-study-act), which aims to propose improvement cycles for units^20^, were also employed. A strategy PlanificaSUS uses refers to prioritizing the implementation of these instruments in laboratory units, PHC and SOC spaces that could full implement of HCP (by having, e.g., multidisciplinary teams and a good physical structure). These centers would function as a showcase for the theoretical-operational learning of PlanificaSUS in their respective health regions, configuring references for other municipalities^13^. The proposal is based on the technical support of the project team to the technical-managerial staff of the state and municipal health departments to plan, organize, and operationalize workshops and teaching activities with PHC and SOC professionals. The project also aims to train tutors to conduct activities in their regions and prepare technical notes, guides, and other initiatives that reach the network as a whole^19,20^.

Fieldwork was carried out in the municipalities of each health region and in at least one small municipality that depended on regional specialized health services. This municipality was chosen based on some health indicators and the team of researchers, later validated by technicians from the regional structures of the respective SHS.

After this agreement, pairs of researchers went to the field to conduct interviews with SHS (such as central and regional management technicians), Municipal Health Departments (MHS) in the chosen municipalities (such as management technicians and professionals from the UBS-Laboratory), and SOC social/institutional actors.

Direct observation of the health services involved in the project (PHC units called UBS-Lab and SOC) was also used. The researchers also participated in regional interagency commission (RIC) meetings to find the political and technical role of SHS and municipality representatives.

The Planifica project was proposed to take place in four stages: 1) Prior planning; 2) Presentation and articulation with state entities; 3) Workshops and seminars; and 4) Implementation of macro-processes and improvement cycles^19,20^. Fieldwork occurred when the municipalities were in stage four of Planifica, which sought to implement improvement processes for PHC and SOC, including dispersion activities and technical-managerial support to state and municipal management in SOC and proposing control plans.

The first fieldwork was carried out from September 2019 to February 2020. This research planned two face-to-face moments in the field but the evolution of the COVID-19 pandemic in Brazil changed its initial schedule. Thus, the second stage was restricted to individual or collective online interviews from August to October 2020. In total, 80 actors in the four health regions were interviewed in the first stage and 51 in the second stage of this study (Chart 1).

**Chart 1:** Interviews conducted with social/institutional actors from four health regions by federative unit, institutional link, and stage of the study (2019 and 2020).

Institutional affiliation of the interviewee	Health Regions									

	BA		MT		PE		RS		Total	
				
	Stage 1	Stage 2	Stage 1	Stage 2	Stage 1	Stage 2	Stage 1	Stage 2	Stage 1	Stage 2
SHS Management										
Central	1	2	2	3	6	4	2	5	11	14
Regional	-	-	4	-	5	5	5	5	14	10
MHS Management										
Large municipality	3	1	3	2	3	3	4	1	13	7
Small municipality	4	1	2	1	3	5	1	1	10	8
UBS-Lab										
Large municipality	3	-	1	1	3	3	2	1	9	5
Small municipality	5	-	2	-	3	2	2	2	12	4
Specialized care outpatient clinic	1	-	2	1	5	2	3	-	11	3
Total	17	4	16	8	28	24	19	15	80	51

SHS: State Secretariat of Health; MHS: Municipal Health Secretary; UBS: basic health unit; Mun.: municipality; BA: Bahia; MT: Mato Grosso; PE: Pernambuco; RS: Rio Grande do Sul.

These open and in-depth interviews were triggered by the following question: “Tell us about how you do your job.” If the need to further develop the themes this research considered central arose during the interviews (articulation of PHC with SOC and attention to tracer conditions), interviewees were provoked to address them.

The theoretical framework used in data analysis refers to implementation science, a field of study that uses methods and techniques to systematically use scientific evidence in public policies to improve the quality and effectiveness of health practices and services^35,36^.

For this, three categories or analytical dimensions were adapted from the conceptual matrix from the Consolidated Framework for Implementation Research^36^:

### Politics (and social actors)

Existing power relations, those built in the process of implementation of HCP by PlanificaSUS, and involved social/institutional actors, including the questions: “What are the power relations between the state and municipal levels?” and “How do political actors and technicians get involved in the process?”

### Technical-operational

Strategies, tactics, and management tools applied in the PlanificaSUS process to implement HCP. What strategies, tactics, and management instruments proposed by PlanificaSUS are being implemented?

### Internal and external contexts

The interrelation of circumstances in health regions and municipalities that affect or may influence the PlanificaSUS process to implement HCP, and especially, how are context variables being considered in implementation?

The three dimensions were analyzed in an articulated and multilevel manner considering the municipal, regional, and state scales; PHC and SOC; and internal and external contexts (Chart 2).

**Chart 2:** Analytical dimensions with management areas and internal and external contexts.

Prism		
Policies (and social actors)	Operational-Technical	Context
State level	PHC	Internal
Regional level	SOC	External
Municipal level	Regulation of access to SOC	

SOC: specialized outpatient care; PHC: primary health care.

Source: adapted from Keith et al.^36^ (2017).

Analysis involved transcribing the recorded interviews, carefully reading them, and finding the emerging patterns, themes, or categories that dialogued with the analytical matrix. Content that pointed to barriers and facilitators were apprehended from the empirical material in interviewees’ statements, understood as elements of the political, technical-operational, or context fields that would enable the analysis of the facilities and difficulties of implementing the analyzed process.

This research was approved by the Research Ethics Committee of the School of Public Health at Universidade de São Paulo (CEP Opinion no. 5.421.751, CAAE 18875719.0.0000.5421). Informed consent forms were signed by interviewees and all the recommended ethical recommendations were followed.

## RESULTS AND DISCUSSION:

### Political Dimension

The political dimension shows harmonious and conflictual relations between federated entities, characterizing the Brazilian federalism as sometimes cooperative and, at others, conflicting^37^. Chart 3 shows the facilitating (in the macropolitical, local-regional-state, regional, and municipal levels) and hindering elements to the achievement of the PlanificaSUS Project.

**Chart 3:** Political dimension.

Facilitating factors	
Political support from SHS	SHS Central Level Engaged
	Existence of projects for the organization of specialized care under SHS management prior to PlanificaSUS—participation of primary care coordination or advisory
	The SHS institutional support strategy enabled the systematic monitoring of planning implementation
Inter-municipal consortium	Political valorization of the consortium format
	Initiatives to create regional health polyclinics with the participation of municipalities
SHS Regional Management	An important political actor supporting municipalities but with different weight across regions
Municipal managers of SUS	They believe that PlanificaSUS improves PHC and, in a way, facilitates access to SOC
Small municipalities	Important political actors to reorganize work processes in PHC
	Maintained consistent adherence to PlanificaSUS
Hindering elements	
Conflicts in secondary level organization	Conflicting interests between civil servants and the SHS regional structure
Tensions between MHS and SHS	Insufficient funding, lack of transfers of resources (from SHS to MHS)
	Different forms of management of SOC, in general, with a greater leading role of the state entity
Tensions with private (philanthropic) providers	Locoregional divergences on the organization and implementation of SOC (autonomy of providers without public management regulation)
	Conflicting relationship between MHS and private provider hospitals (Santas Casas) in SOC management
	Contractualization by central SHS with little or no regional participation hinders the construction/agreement of a regional SOC project
Fragility of collegiate bodies	Fragility of the RIC Technical Chambers
Constant change of municipal and state managers	Frequent change of managers and management guidelines in municipalities weakened in agreements
	Change of central and regional state SUS managers

PHC: primary health care; SOC: specialized outpatient care; SHS: State Health Secretariat; MHS: Municipal Health Secretariat; SUS: Unified Health System; RIC: Regional Interagency Commission.

A central element in the process is the leading role of states, which has grown since the beginning of the 21st century, breaking with the design of decentralization-regionalization focused on the municipality that characterized the first decades of SUS^38,39^.

Most regions had a state political project to expand access to SOC and organize it in articulation with the PHC. This context valued and incorporated PlanificaSUS as an organizing axis of this process since it would support municipalities, strengthen PHC, improve and rationalize the sharing of care with SOC.

In the regions which implemented them, managers highly valued intermunicipal consortia, especially in small municipalities as they more greatly provided specialty consultations within their scope of management^40,41^. Consortia contributed to reduce local shortcomings such as scarce financial, technological, and human resources to implement public policies^40^. The balance between demand and supply of SOC services considering the population-based management of HCN is a central point for the PlanificaSUS organizational design. The strategy of intermunicipal consortia also produced a vector of facilitation due to the stronger regional logic from such articulations and increased the bargaining power of the municipalities involved with the state government, guaranteeing resources to the region that would be difficult to obtain if requested in isolation^40^.

In this case, the adherence of the state management to the HCP methodology is one of the most important political elements since the project is unable to start without SHS endorsement and support. It also contributes to project feasibility based on the choice of regions for implementation and the support of their regional structures, guiding the project in RIC and bipartite intermanagement commissions, indicating it as a strategy for regional planning and articulation^42^. Notably, the regional level configures a distinct decision-making space in the studied regions, showing the heterogeneous construction of regional management space in Brazil. However, some regions showed that the support of state management to PlanificaSUS limited itself to PHC advisory and coordination without the necessary involvement of other strategic sectors included in the construction of the HCN.

Our findings reinforce that a great challenge to consolidate SUS refers to strengthening the articulation of federated entities and the leading role of intergovernmental forums. State power spheres still often face difficulties to effectively coordinate this process, highlighting the weaknesses of the political-administrative construction of Regional Management Collegiates, which make them more vulnerable to private interests. However, the analyzed process showed the leading role of the state and the tensions between municipal and state managers, particularly on the non-transfer of resources by the SHS, which weakened bipartite intermanagement commissions by “blocking” and emptying agendas. The RIC at the studied regions often lacked technical chambers that could support processes of discussion and agreement for the organization of networks and lines of care; nevertheless, they guided the discussion on PlanificaSUS. Regionalization, more than a process of organizing health actions and services in the territory to ensure comprehensive care, is a political construction that should favor dialogue between local actors and federate managers to find and face the health needs of specific territories^43^.

Smaller municipalities stood out as political actors with the greatest role in facilitating the implementation of PlanificaSUS. Despite heavily depending on regional services, they made better use of all the elements to reorganize PHC^39^, and their management teams strive, as far as possible, to guarantee access to health services, corroborating Pinafo et al.^39^ (2020).

In the studied regions, frequent changes in municipal managers and management priorities and state SUS technicians and managers weakened regional agreement processes, constituting a culture of transience toward projects under implementation.

On the other hand, tensions related to the co-financing of specialized services, state transfers of inputs, and agreement of resources between municipal administrations and the SHS acted as factors that hindered the implementation of the HCP. They also highlighted some conflicts between hub municipalities (local headquarters) and those disputing resources, service offers, and power. Health governance involves power relations and is constantly stressed by the interaction between the subjects organizing state policies (expressed in RIC meetings), as per Nogueira et al.^42^

One political barrier to implementation relates to the fragmented logic of providers and the corporate power of some professional categories that strain network organization. Thus, the power of specialists must always be considered in the construction of coping strategies. In the studied regions, this issue was more evident in the organization of the maternal and child health care line based on tensions between obstetrician gynecologists, who disputed the SOC organization model. As per Ribeiro et al.^38^, it is imperative to establish genuine regional governance with the state manager playing a leading role.

Tensions with private providers, particularly *Santas Casas* (which are responsible for SOC in some of the studied regions), have also hindered the implementation of lines of care. This study found that, despite being contracted, these services act with great autonomy and little regulation by the public entity and that regional SHS structures played a limited role in agreements and control of contracted services.

### Technical-operational dimension

The main facilitating elements in this dimension relates to the involvement of service professionals, the incorporation of some PlanificaSUS tools, and the lessons learned from the pandemic. Hindering elements include the lack of a provision for a budget for expenses inherent to PlanificaSUS and the low involvement of sectors outside SHS (Chart 4).

**Chart 4:** Technical-operational dimension.

Facilitating factors	
Involvement of service professionals	Valuing capillary planning among professionals
Incorporation of PlanificaSUS tools	Planning tools built into local plans
	Incorporation into PHC work processes
	Use of planning instruments in PHC care activities
Hindering elements	
Insufficient budget forecasting for all needs	Unforeseen costs to develop PlanificaSUS hindered the participation of several municipalities
	Lack of operational alternatives to adapt to the project
Lack of flexibility in the PlanificaSUS model	Strategies to implement PlanificaSUS: barely open to locoregional adaptations
	Rigidity in task control
Less involvement of other areas of management in the operation of the project	Little involvement of other strategic sectors of the SES beyond primary care

PHC: primary health care; SUS: Unified Health System.

As previously mentioned, small municipality management valued the training provided by PlanificaSUS, pointing out its importance as a strategy to reorganize PHC services. Larger municipalities adhered to central teams and especially laboratory unit teams, which changed work dynamics and multiplied, in addition to these units, all the tools implemented and adopted in the articulation of PHC with SOC.

The units that participated in PlanificaSUS incorporated some of the HCP tools the action plan supports into the daily routine of their services, such as dashboards and PDSA. They valued a culture of planning and monitoring, with some local plans promoting PHC reorganization, such as registration task forces, staggered scheduling in shifts, and family risk classification. Other studies have also found that HCP has contributed to improving PHC processes, including user registrations and e-SUS records, better organization of the flows of users and professionals at UBS, and greater integration of professionals^12^.

PlanificaSUS provided the teams with traditional management tools that services failed to use daily basis. The incorporation of these practices contributes to organize and improve PHC and the care for the population. More than a change in the logic of management, PlanificaSUS added useful technologies that expanded teams’ toolbox. Some processes stood out, such as: (1) population registration, (2) risk stratification, (3) flow organization, (4) appointment scheduling, and (5) digitalization. Some authors confirm that changes in structures and especially processes should occur in PHC to socially build a care coordination model^19^.

The balanced presence of general practitioners and specialists is essential from the perspective of health care systems structured in HCN, acting together to benefit users^20^.

Therefore, from the technical-operational perspective, consensus among professionals, new management tools incorporated into services, and new learning curves strengthen the articulation between PHC and SOC. Evangelista et al.^12^ found that continuing education broadened the field of action of professionals, improved actions at both levels of care, and consolidated shared care, with the possibility of mutual adjustment and technical support between both teams.

The unforeseen operational and infrastructure costs of carrying out PlanificaSUS activities represented a tension that hindered the participation of several municipalities.

The constant change of tutors and the lack of access to computerized networks also configured factors that weakened the preparation and strength of meetings, leading to less involvement from participants and its repercussions on the teaching-learning process.

Another complicating element that emerged among participants relates to the model PlanificaSUS adopted to implement HCP as it executes activities in preparatory, operational, and control stages following a schedule. This very structured proposal has few openings for flexibility and adaptation to local realities. Interviewees also reported excessive and rigid tasks that ignore service dynamics and their limited deadline.

### Context Dimension

Facilitating context factors relate to the expertise of state teams in HCP and in the implementation of the analyzed lines of care. The organization and articulation of municipal managers in the regional constitution in some territories is also of note. Chart 5 shows the main facilitating and hindering elements in this dimension.

**Chart 5:** Context Dimension

Facilitating factors	
Cumulative frequency	Some articulation initiatives between PHC and SOC
	Existing lines of care and those being implemented in the regions, facilitating adherence
	SHS previous planning experience
Organization and articulation of municipal managers	Prior articulation of local municipalities
	Prior sharing of services and funding
Hindering elements	
Management of SUS work (SHS and MHS)	Frequent losses of health workers in care teams (administrative reform; precarious employment contracts; extinction of the More Doctors Program; COVID-19 pandemic)
	The pandemic has completely changed the work processes of laboratory units and the secondary level
Relationship between MHS, SHS, and MH	Municipalities found insufficient support from the SHS to implement lines of care
	Changes in the national PHC policy impacted PlanificaSUS
Pandemic	Increased PHC workload and occasional closure of SOC
	Constant absence of professionals

PHC: primary health care; SOC: specialized outpatient care; SUS: Unified Health System; PHC: primary care; SHS: State Health Secretariat; MHS: Municipal Health Secretariat; MH: Ministry of Health.

Internal and external context variables strongly influenced local and regional actions for actors trained in planning and action in locoregional actions. However, the great absence of services or management professionals (as during the pandemic) or the lack of regional coordination cause immense losses to PHC/SOC articulation.

Even so, these variables were diversified and specific in view of the social construction of each region. The premise of PlanificaSUS refers to their specialized outpatient service in the line of care for each region. This type of equipment would offer concrete comprehensiveness in health regions. Our first visit found a history of implementation of these services in host municipalities/hubs that involved local municipalities.

In contrast to facilitating factors, we also found hindering elements regarding the contextual dimension related to the MHS and SHS management of work in SUS. Participants reported several problems that directly impacted the loss of the municipal and state SUS workforce, such as administrative reforms, precarious employment contracts, extinction of the More Doctors Program, and the COVID-19 pandemic.

Finally, we highlight that local and regional managers found that some dissonances in the management of federated entities impacted HCP implementation. Municipal managers showed apprehension about the effective support from SHS in this process as they reported experiences in which such institutional support were unable to construct care lines. Another tension relates to the impacts of the changes by the Ministry of Health to the National Primary Care Policy and the More Doctors program. These changes would generate losses of resources for municipalities and the need to review their priorities, which directly impacted the continuity of the HCP implementation process following PlanificaSUS.

The Figure synthesizes the discussed results and points out the importance of process continuity since the pandemic has redirected the decisions and operations of the health system to the care of acute conditions, which leaves chronic conditions with an even greater need for articulation to guarantee full access to PHC, SOC, and HCN^44^.

**Figure:**
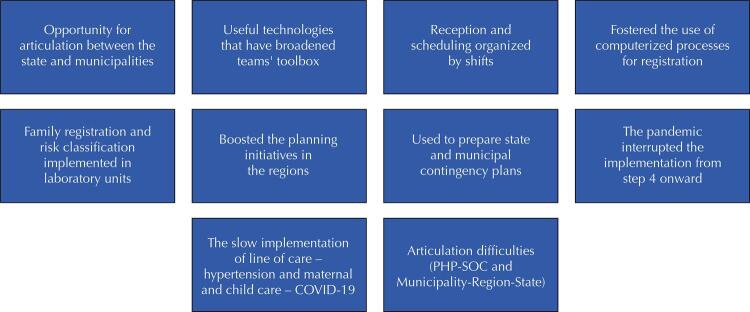
Aggregated synthesis of results by three analytical dimensions.

PHC: primary health care; SC: specialized outpatient care; FHS:

In addition to the demands of COVID-19, people with chronic (acute or not) or non-COVID-19 acute conditions bring important challenges to SUS managers.

## CONCLUSION

This study evaluated the HCP PlanificaSUS project implementation process in four pre-selected health regions.

These regions showed the characteristics of the health care process regarding the implementation of new practices and the contextual variables that influence technical-political actions in health.

Results indicated important facilitators and hindrances for the implementation of the HCN, initiated by the qualification of PHC and SOC in the four studied regions.

It was notorious that many regions used the PlanificaSUS project as an opportunity to articulate states and municipalities and place an important political bet to build networks and lines of care, but many paths are to be undertaken. It is important to consider the role of state managers in central and regional investments related to the project and the effect of PHC mobilization processes and expansion of its power, especially in small municipalities.

Thus, it is important to point out that a culture of local planning has been established in PHC, even in unfavorable contexts, but often restricted only to laboratory units. There remains a long way to an articulated regional planning with specialized care that goes beyond distributing vacancies across municipalities.

Based on the framework of the social construction of PHC^13^, it is possible to make an analysis based on HCP macro and micro processes. In other words, SUS, a system based on PHC and accordingly organized that permanently seeks to improve care, continues to be a permanent HCP agenda. The project contributed to PHC routinely facing its capacity to ensure the continuity of care among its various services.

As a possible result of this process, we seek to improve the structure for PHC care and implement all the macro-processes established in the PHC intervention model.
