# Supplementary material for: Challenges to implementing planning processes in Brazilian health regions
Source: Rev Saude Publica. 2024 Apr 1;57(Suppl 3):2s. doi: 10.11606/s1518-8787.2023057005138 (PMC11037912; doi:10.11606/s1518-8787.2023057005138)
Supplement: Translation

# Translation: Desafios para a implementação de processos de planificação em regiões de saúde

**Authors:** Oswaldo Yoshimi Tanaka, Marco Akerman, Marília Cristina Prado Louvison, Aylene Bousquat, Nicanor Rodrigues da Silva Pinto, Ana Lígia Passos Meira, Lídia Pereira da Silva Godoi, Ana Paula Chancharulo e Morais Pereira, Sandra Maria Spedo, Monique Batista de Oliveira, Ilana Eshriqui, Marcio Anderson Cardozo Paresque

**Affiliations:** I Brasil Universidade de São Paulo. Faculdade de Saúde Pública. São Paulo, SP, Brasil; II Brasil Universidade Federal de São Paulo. Programa de Pós-Graduação em Saúde da Família. São Paulo, SP, Brasil; III Brasil Universidade do Estado da Bahia. Departamento de Ciências da Vida. Salvador, BA, Brasil; IV Brasil Hospital Israelita Albert Einstein. Centro de Estudos Pesquisa e Prática em Atenção Primária à Saúde e Redes. São Paulo, SP, Brasil

**Keywords:** Atenção Primária à Saúde, Regionalização da Saúde, Apoio ao Planejamento em Saúde

## Abstract

**OBJETIVO:**

Reconhecer elementos que facilitaram ou dificultaram as etapas do processo de implementação do PlanificaSUS.

**MÉTODOS:**

Foi realizado um estudo de casos múltiplos em quatro regiões de saúde pré-selecionadas – Belo Jardim (PE), Fronteira Oeste (RS), Sul-Mato-Grossense (MT), Valença (BA) –, utilizando como condições traçadoras a hipertensão arterial sistêmica e a atenção materno infantil. Foi realizada observação participante na Comissão Intergestora Regional (CIR), bem como entrevistas em profundidade nas quatro regiões estudadas, com informantes-chave da gestão estadual e municipal e com profissionais dos serviços da atenção primária à saúde e atenção ambulatorial especializada envolvidos no projeto. Construiu-se análise em três dimensões: política, técnico operacional e contexto.

**RESULTADOS:**

Na dimensão política, ficou evidenciado que, de alguma forma, as regiões identificaram no projeto uma oportunidade de articulação entre estados e municípios e uma aposta política importante para a construção de redes e linhas de cuidado, mas ainda com muitos caminhos a percorrer para enfrentar as barreiras identificadas nas disputas em jogo na construção do Sistema Único de Saúde (SUS). Na dimensão técnico operacional, é importante considerar que foi estimulada uma cultura de planejamento local na APS, além do favorecimento do uso de ferramentas tradicionais, no sentido de organizá-la e qualificá-la, como a organização dos cadastros, da agenda e da demanda. No entanto, os processos centralizados formativos e indutores da planificação nem sempre respondem às necessidades singulares dos territórios e podem produzir barreiras à implementação.

**CONCLUSÕES:**

Cabe considerar o papel do gestor estadual na aposta relacionada ao projeto, tanto no nível central quanto no nível regional, e o efeito de processos de mobilização da atenção primária à saúde e amplificação de sua potência, mas ainda com muitos caminhos a percorrer para enfrentar as barreiras identificadas nas disputas em jogo na construção do SUS.

## INTRODUÇÃO

Os sistemas de saúde, em especial aqueles alicerçados na concepção da saúde como um direito humano, enfrentam inúmeros e crescentes desafios. Certamente, um dos maiores é o de conseguir responder ao perfil de adoecimento das populações, decorrente da transição demográfica e epidemiológica. Vale ressaltar que as trajetórias de alterações e ajustes nos sistemas de saúde não se deram de modo linear, transitando desde propostas inovadoras até meros ajustes técnicos e gerenciais^1^.

No Brasil, a situação de saúde caracterizada pela tripla carga de doença, o acelerado envelhecimento populacional e a fragmentação assistencial também representam desafios para o Sistema Único de Saúde (SUS). Nesse sentido, estratégias que promovam a integração assistencial e modificações nos processos de trabalho em saúde (em especial no manejo das condições crônicas) configuram-se como essenciais para garantir a qualidade do cuidado e a melhoria do desempenho de um sistema de saúde^2^que se pretende equânime, universal e integral.

A articulação dos serviços de saúde é fundamental para garantia da integralidade e a continuidade do cuidado^3^, com destaque para a integração entre a atenção ambulatorial especializada (AAE) e a atenção primária à saúde (APS), que tem sido considerada um ponto nevrálgico do sistema^4^. Esta integração não se resume a um simples procedimento administrativo, tanto é que os resultados alcançados são bastante distintos entre os países.

No caso brasileiro, a proposta de alcançar a integralidade, garantindo a continuidade do cuidado, se alicerçou na constituição de redes de atenção à saúde (RAS) regionalizadas e concebeu a APS como o centro ordenador e coordenador do cuidado, articulado com a oferta da AAE. Não obstante, esta proposta esbarra na insuficiência de oferta, na heterogeneidade da distribuição territorial e na forma de organização dos serviços de AAE^5^.

Na tentativa de mudar tal cenário, experiências que fomentem a integração da APS e AAE têm sido implementadas no Brasil e mundialmente^1,11^. Dessa forma, a análise delas é crucial para o entendimento dos complexos processos de construção da RAS. No caso brasileiro, uma das experiências que se destaca é a Planificação de Atenção à Saúde (PAS), que foi proposta pelo Conselho Nacional de Secretários de Saúde (Conass) como uma metodologia para a organização de serviços e integração de RAS^14^. Inicialmente, as ações da PAS, até então denominada “Planificação da Atenção Primária à Saúde nos Estados”, direcionavam-se à APS e tinham foco na gestão^16^. A partir do delineamento das diretrizes das RAS, a AAE passa a ser incluída no processo^17^.

Dentro das propostas de PAS implementadas no país, pelo seu alcance e número de atores envolvidos, se destaca o “PlanificaSUS”, que se trata de um projeto do Programa de Apoio ao Desenvolvimento Institucional do Sistema Único de Saúde (Proadi-SUS), desenvolvido pelo Hospital Israelita Albert Einstein, em parceria com o Conass.

O primeiro triênio do PlanificaSUS (2018–2020) foi executado em 26 regiões de saúde do país, com o objetivo de implantar a PAS, fortalecendo a APS e sua organização em rede com a AAE^18^. Nesse sentido, este artigo tem como objetivo principal analisar a experiência de implantação, identificando elementos dificultadores e facilitadores e tomando como referência a integração APS-AAE^17,21^.

## MÉTODOS

Foi realizado um estudo de casos múltiplos^22,23^em quatro regiões de saúde do país, escolhidas a partir de um total de 27 (na ocasião da escolha, houve depois desistência de uma região), que participavam do projeto PlanificaSUS, entre 2019 e 2020. Essa escolha foi realizada de forma intencional pelos pesquisadores e corroborada pela coordenação do projeto. Considerando o estado de desenvolvimento e existência de um município escolhido como a sede do projeto em cada estado, bem como local das unidades laboratórios e serviços onde as ações do PlanificaSUS estavam sendo desenvolvidas, definiu-se seguirmos com o estudo nestes municípios polos e com outro da região, onde só tivessem à disposição os serviços de atenção básica e dependesse da AAE de município polo.

Inicialmente, foram definidos 56 indicadores, coletados das bases de dados do sistema Datasus e do Sistema da Pesquisa Região e Redes: Caminho da Universalização da Saúde no Brasil^24^. Esses indicadores contemplaram: oferta de serviços; produção de atividades ambulatoriais; produção de atividades hospitalares; nascidos vivos; e taxa de mortalidade por causa.

Os dados de estrutura, processo e resultado foram coletados e analisados quanto a coerência, tendência e relações entre atividade de distintas complexidades de atendimento. Tais variáveis foram analisadas de acordo com as linhas de cuidado prioritárias de atenção materno-infantil (Rede Cegonha)^25^e de atenção à hipertensão e diabetes, escolhidas pela sua importância no cenário epidemiológico brasileiro^29^.

Com base na análise desses indicadores, foram pré-selecionadas 12 regiões de saúde que apresentavam contradições entre oferta de serviços de saúde e indicadores de morbimortalidade. Essas contradições teriam potencial de contribuir para desvendar características do processo de atenção à saúde e identificar variáveis de contexto, que influenciam na implementação de ações e políticas de saúde.

A escolha final das regiões de saúde foi realizada em uma oficina de trabalho, além de envolver a equipe de pesquisadores e a equipe coordenadora do projeto PlanificaSUS, esta última ajudou no direcionamento das escolhas por conhecê-las melhor, considerando as características políticas, operacionais e de contexto. Nesse processo, foram selecionadas quatro regiões de saúde de distintas unidades federativas do país (Bahia, Mato Grosso, Pernambuco e Rio Grande do Sul). Optou-se por compor os casos para estudo com duas regiões que priorizavam, para o projeto PlanificaSUS, a linha de cuidado de atenção à saúde materna e infantil (MT e RS) e outras duas que priorizavam a linha de cuidado de atenção à saúde das pessoas com hipertensão e diabetes (BA e PE),

Para cada região escolhida, foi elaborada uma matriz avaliativa e, a partir de informações disponíveis, foram identificados informantes-chave para as entrevistas de campo.

O planejamento do trabalho de campo da pesquisa foi pactuado em reuniões remotas com interlocutores das Secretarias Estaduais de Saúde (SES), que atuavam como referência técnica para o projeto PlanificaSUS nas unidades federativas selecionadas. O desenho da pesquisa dialogou com as principais características e objetivos do projeto, que busca integrar e fortalecer macro e micro processos da APS e integrá-la em rede com a AAE. Para isso, utiliza instrumentos específicos da PAS, tais como o *dashboard* (painel de bordo), que permite a visualização rápida de indicadores para monitoramento do desempenho cotidiano das equipes^13^. Também aplica metodologias de gerenciamento de processos, como o PDSA, sigla em inglês para planejamento, execução (do), estudar (*study)* e ação corretiva, que tem o objetivo de propor ciclos de melhoria nas unidades^20^. Uma das estratégias utilizadas pelo PlanificaSUS foi a priorização de implementação destes instrumentos em unidades laboratório, espaços de APS e AAE que possuíam condições para a plena implementação da PAS (como equipes multiprofissionais e boa estrutura física). Esses centros funcionariam como vitrine para o aprendizado teórico-operacional do PlanificaSUS em suas respectivas regiões de saúde, caracterizando-se como referências para outros municípios^13^. A proposta se assenta no apoio técnico da equipe do projeto ao corpo técnico-gerencial das secretarias estaduais e municipais de saúde para o planejamento, organização e operacionalização de workshops e oficinas tutorais, envolvendo os profissionais da APS e AAE. O projeto atua também nas perspectivas de formação de tutores para condução das atividades em suas regiões e elaboração de notas técnicas, guias e outras iniciativas que alcançam o conjunto da rede^19,20^.

O trabalho de campo foi realizado nos municípios sede de cada região de saúde e, em pelo menos um município de pequeno porte, dependente de serviços de saúde especializados de abrangência regional. A escolha desse município foi realizada a partir de alguns indicadores de saúde, pela equipe de pesquisadores e, posteriormente, validada por técnicos das estruturas regionais das respectivas SES.

Após esta pactuação, os pesquisadores, em duplas, foram a campo para a realização das entrevistas com atores sociais/institucionais vinculados a: SES (técnicos da gestão dos níveis central e regional); Secretaria Municipal de Saúde (SMS) dos municípios selecionados (técnicos da gestão e profissionais das UBS-Laboratório) e AAE.

Outra técnica utilizada foi a observação direta dos serviços de saúde envolvidos no projeto: as unidades de APS, chamadas de UBS-Lab, e os AAE. Houve, ainda, a participação dos pesquisadores em reuniões das Comissões Intergestores Regional (CIR), visando a identificar o papel político e técnico dos representantes da SES e dos municípios.

O projeto Planifica foi proposto para ocorrer em quatro etapas: 1) Planejamento prévio; 2) Fase de apresentação e articulação com os entes estaduais; 3) Oficinas e seminário; e 4) Implantação dos macroprocessos e ciclos de melhoria^19,20^. A atividade campo ocorreu quando os municípios estavam na etapa 4 do Planifica, que buscava a implantação dos processos de melhoria na APS e na AAE, com atividades de dispersão e apoio técnico-gerencial a gestão estadual e municipal na AAE e com a proposta do plano de controle.

O primeiro momento do campo foi realizado entre setembro de 2019 e fevereiro de 2020. A pesquisa previa dois momentos presenciais em campo, contudo, em decorrência da evolução da pandemia da covid-19 no país, impôs-se mudanças na programação inicial. Nesse sentido, a segunda etapa ficou restrita a entrevistas online, realizadas individual ou coletivamente, no período de agosto a outubro de 2020. Os atores entrevistados, nas quatro regiões de saúde, totalizaram 80 na primeira etapa e 51 na segunda etapa do estudo (Quadro 1).

**Quadro 1:** Entrevistas realizadas com atores sociais/institucionais das quatro regiões de saúde por unidade federativa, vínculo institucional e etapa do estudo (2019 e 2020).

Vínculo institucional da(o) entrevistada(o)	Regiões de saúde									

	BA		MT		PE		RS		Total	
				
	1ª etapa	2ª etapa	1ª etapa	2ª etapa	1ª etapa	2ª etapa	1ª etapa	2ª etapa	1ª etapa	2ª etapa
Gestão SES										
Central	1	2	2	3	6	4	2	5	11	14
Regional	-	-	4	-	5	5	5	5	14	10
Gestão SMS										
Município grande	3	1	3	2	3	3	4	1	13	7
Município pequeno	4	1	2	1	3	5	1	1	10	8
UBS-Lab										
Município grande	3	-	1	1	3	3	2	1	9	5
Município pequeno	5	-	2	-	3	2	2	2	12	4
Ambulatório de atenção especializada	1	-	2	1	5	2	3	-	11	3
Total	17	4	16	8	28	24	19	15	80	51

SES: Secretária Estadual de Saúde; SMS: Secretária Municipal de Saúde; UBS: unidade básica de saúde; Mun.: município; BA: Bahia; MT: Mato Grosso; PE: Pernambuco; RS: Rio Grande do Sul.

As entrevistas foram abertas e em profundidade, desencadeadas pela pergunta disparadora: “Conte-nos sobre como realiza seu trabalho?”. No decorrer das entrevistas, quando se identificava a necessidade de aprofundamento de temas considerados centrais para a pesquisa (articulação da APS com a AAE; e atenção às condições traçadoras), os entrevistados foram provocados a abordá-los.

Na análise dos dados, o referencial teórico utilizado foi o da ciência da implementação, que se caracteriza como um campo de estudos que utiliza métodos e técnicas para impulsionar o uso sistemático de evidências científicas em políticas públicas, visando a aprimoração da qualidade e a efetividade de práticas e serviços de saúde^35,36^.

Para tanto, foram selecionadas três categorias ou dimensões analíticas adaptadas da matriz conceitual do *Consolidated Framework for Implementation Research* (CFIR)^36^:

### Política (e atores sociais)

Relações de poder existentes e as construídas no processo de implementação da PAS pelo PlanificaSUS, além dos atores sociais/institucionais envolvidos. Incluindo as questões de: “Como se apresentam as relações de poder entre o nível estadual e municipal?” e de “Como os atores políticos e os técnicos se envolvem no processo?”.

### Técnico operacional

Estratégias, táticas e ferramentas de gestão aplicadas no processo de implementação da PAS pelo PlanificaSUS. Quais estratégias, táticas e instrumentos de gestão, propostos pelo PlanificaSUS, estão sendo implementados?

### Contextos (internos e externos)

Interrelação de circunstâncias presentes, nas regiões de saúde e nos municípios, que afetam ou podem afetar o processo de implementação da PAS pelo PlanificaSUS. E, especialmente, como as variáveis de contexto estão sendo levadas em conta na implementação?

As três dimensões foram analisadas de forma articulada e multinível, considerando: as escalas municipal, regional e estadual; a APS e a AAE; além dos contextos internos e externos (Quadro 2).

**Quadro 2:** Dimensões analíticas com os âmbitos de gestão e os contextos interno e externo.

Dimensões		
Política – atores sociais	Técnico-operacional	Contexto
Nível estadual	APS	Interno
Nível regional	AAE	Externo
Nível municipal	Regulação do acesso à AAE	

AAE: atenção ambulatorial especializada; APS: atenção primária à saúde.

Fonte: adaptado de Keith et al.^36^ (2017).

O processo de análise envolveu a transcrição das entrevistas gravadas, seguida de leitura detalhada e identificação de padrões, temas ou categorias emergentes que dialogassem com a matriz analítica. A identificação de conteúdos, nas falas dos entrevistados, que apontassem para as barreiras e os facilitadores foram apreendidas do material empírico produzido, compreendendo ainda se tratar de elementos do campo da política, do técnico operacional ou do contexto que permitissem analisar as facilidades e dificuldades da implementação do processo analisado.

A pesquisa foi aprovada pelo Comitê de Ética em Pesquisa da Faculdade de Saúde Pública da USP (Parecer CEP nº 5.421.751, CAAE18875719.0.0000.5421). Os participantes entrevistados assinaram o TCLE e foram seguidas todas as recomendações éticas preconizadas.

## RESULTADOS E DISCUSSÃO

### Dimensão Política

Na dimensão política, é possível observar tanto relações harmoniosas quanto conflituosas entre os entes federados, o que é traço característico do federalismo brasileiro que, por vezes, assume feição cooperativa e, em outras, o conflito é a tônica^37^. Os elementos facilitadores relacionados no âmbito da macropolítica e ao nível loco regional-estadual, regional e municipal e os elementos dificultadores para a consecução do Projeto PlanificaSUS podem ser visualizados no Quadro 3.

**Quadro 3:** Dimensão política.

Elementos facilitadores	
Respaldo político da SES	Nível central da SES engajado
	Existência de projetos de organização da atenção especializada, sob gestão da SES, previamente ao PlanificaSUS- participação da coordenação ou assessoria da atenção básica
	Estratégia de apoio institucional da SES possibilitou acompanhamento sistemático da implementação da planificação
Consórcio intermunicipal	Valorização política do formato consórcio
	Iniciativas de criação policlínicas regionais de saúde com participação de municípios
Gerência Regional da SES	Importante ator político, dando apoio aos municípios, mas com peso distinto nas diferentes regiões
Gestores municipais do SUS	Acreditam que PlanificaSUS qualifica a APS e de certa forma facilita o acesso a AAE
Municípios de pequeno porte	Atores políticos importantes para reorganizar os processos de trabalho na APS
	Mantiveram adesão consistente ao PlanificaSUS
Elementos dificultadores	
Conflitos na organização do nível secundário	Interesse conflitantes dos servidores e estrutura regional da SES
Tensões entre SMS e SES	Financiamento insuficiente, falta de repasses de recursos (da SES para SMS)
	Distintas formas de gestão da AAE, no geral, com maior protagonismo do ente estadual
Tensões com prestador privado (filantrópico)	Divergências locorregionais sobre a organização e implementação do AAE (autonomia do prestador sem regulação da gestão pública)
	Relação conflituosa entre SMS e hospitais prestadores privados (Santas Casas) na gestão da AAE
	Contratualização realizada pelo nível central da SES, com escassa ou nenhuma participação do nível regional, dificulta a construção/pactuação de um projeto regional de AAE
Fragilidade de instâncias colegiadas	Fragilidade de Câmaras Técnicas das CIR
Constantes trocas de gestores (municipais e estaduais)	Mudança frequente de gestores e das diretrizes da gestão nos Municípios, fragilizados nas pactuações
	Troca de gestores do SUS estadual, tanto no nível central quanto no regional

APS: atenção primária à saúde; AAE: atenção ambulatorial especializada; SES: Secretária Estadual de Saúde; SMS: Secretária Municipal de Saúde; SUS: Sistema Único de Saúde; CIR: Comissão Intergestora Regional.

Um elemento central no processo é o protagonismo do nível estadual, que vem crescendo desde o início do século XXI, rompendo o desenho de descentralização-regionalização focado no município que foi característico das primeiras décadas do SUS^38,39^.

Na maior parte das regiões, observou-se a existência de projeto político estadual voltado a ampliar o acesso e organizar a AAE, em articulação com a APS. Nesse contexto, o PlanificaSUS foi valorizado e incorporado como um dos eixos organizadores desse processo, já que cumpriria as funções de apoiar os municípios, fortalecer a APS, qualificar e racionalizar o compartilhamento de cuidado com a AAE.

Nas regiões em que estavam implantados, os consórcios intermunicipais foram muito valorizados pelos gestores, em particular de municípios de pequeno porte, pois lhes proporcionava maior provimento de consultas de especialidades no seu âmbito de gestão^40,41^. A modalidade de consórcio contribuía para a diminuição de carências locais, como a escassez de recursos financeiros, tecnológicos e humanos necessários à implementação das políticas públicas^40^. O equilíbrio entre demanda e oferta de serviços pela AEE, considerando a gestão de base populacional da RAS, é um ponto central para o desenho organizativo proposto pelo PlanificaSUS. A estratégia de consórcios intermunicipais também produziu um vetor de facilitação, considerando que a lógica regional se coloca mais forte a partir de articulações dessa ordem, ademais, aumenta o poder de barganha dos municípios envolvidos junto ao governo estadual, garantindo recursos à região que dificilmente seriam obtidos caso fossem solicitados isoladamente^40^.

Neste caso analisado, a adesão da gestão estadual à metodologia da PAS é um dos elementos políticos mais importantes, visto que, sem o aval e apoio das SES, o projeto sequer se inicia. Contribui também para a viabilização do projeto a partir da escolha das regiões para a implementação, bem como com o apoio de suas estruturas regionais, pautando o projeto nas CIR e CIB, indicando-o como uma estratégia de planejamento e articulação regional^42^. Vale ressaltar que o nível regional se apresenta como um espaço decisório distinto nas regiões estudadas, demonstrando a heterogeneidade da construção do espaço de gestão regional ainda observado no país. Contudo, em algumas regiões, foi observado que o respaldo da gestão estadual ao PlanificaSUS era limitado à assessoria e coordenação da APS, sem o necessário envolvimento de outros setores estratégicos incluídos na construção das RAS.

Os achados desta pesquisa reforçam que um dos grandes desafios para a consolidação do SUS é o fortalecimento da articulação dos entes federados e do protagonismo dos fóruns intergovernamentais. Com frequência, as esferas estaduais de poder ainda apresentam dificuldades para assumir a efetiva coordenação desse processo, merecendo destaque as fragilidades da construção político-administrativa dos Colegiados de Gestão Regional, que os tornam mais vulneráveis aos interesses particulares. No entanto, no processo analisado, o protagonismo estadual foi identificado, bem como as tensões entre gestores municipais e estaduais, particularmente relacionadas ao não repasse de recursos por parte das SES, o que fragilizava a CIB, tanto por “trancamento de pautas” quanto por seu esvaziamento. As CIR das regiões estudadas, muitas vezes, não estavam organizadas com câmaras técnicas que pudessem apoiar processos de discussão e pactuação para a organização das redes e linhas de cuidado, contudo, pautavam a discussão sobre o PlanificaSUS. A regionalização, mais do que um processo de organização das ações e dos serviços de saúde no território, visando assegurar a integralidade da atenção, é uma construção política que deveria favorecer o diálogo entre os atores locais e os gestores federados, para o reconhecimento e enfrentamento das necessidades de saúde dos territórios específicos^43^.

Com relação aos municípios, os de menor porte se destacaram como atores políticos com maior protagonismo na facilitação da implementação do PlanificaSUS. Apesar de fortemente dependentes do serviço regional, aproveitam melhor todos os elementos ofertados para a reorganização da APS^39^, além da equipe gestora se esforçar, na medida do possível, para proporcionar a garantia do acesso aos serviços de saúde, corroborando o apresentado em estudo de Pinafo et al.^39^ (2020).

Nas regiões estudadas, mudanças frequentes de gestores e, consequentemente, de prioridades da gestão nos municípios, bem como de técnicos e gestores do SUS estadual, fragilizavam os processos regionais de pactuação, constituindo uma cultura de transitoriedade em relação aos projetos em implementação.

Por outro lado, tensões entre as gestões municipais e a SES, relacionadas aos processos de cofinanciamento dos serviços especializados, repasses estaduais de insumos e pactuação de recursos, atuavam como fatores dificultadores da implementação da PAS. Destacaram-se, ainda, alguns conflitos entre os municípios polo (sede da região) e os demais na disputa por recursos, ofertas de serviços e poder. A governança da saúde envolve relações de poder, sendo tensionada, o tempo todo, pela interação entre os sujeitos envolvidos na organização da política do estado, expressa nas reuniões da CIR, como referido no estudo de Nogueira et al.^42^

Uma das barreiras para a implementação na dimensão da política está relacionada à lógica fragmentada dos prestadores e o poder corporativo de algumas categorias profissionais que tensionam com a organização da rede. Nesse sentido, o poder dos especialistas precisa ser sempre considerado na construção de estratégias de enfrentamento. Nas regiões estudadas, essa questão ficou mais evidenciada na organização da linha de cuidado materno e infantil, a partir de tensões produzidas pelos ginecologistas obstetras, que disputavam o modelo de organização da AAE. Como clamam Ribeiro et al.^38^, é imperativa a constituição de uma genuína governança regional com protagonismo do gestor estadual.

Tensões com os prestadores privados, particularmente as Santas Casas, responsáveis pela AAE em algumas regiões estudadas, também têm dificultado a implementação de linhas de cuidado. Identificou-se que, apesar de contratualizados, esses serviços atuam com muita autonomia e pouca regulação do ente público. Além disso, constatou-se que as estruturas regionais da SES tinham protagonismo limitado no processo de pactuação e controle dos serviços contratados.

### Dimensão Técnico Operacional

Em relação a esta dimensão, os principais elementos facilitadores identificados estavam relacionados ao envolvimento dos profissionais do serviço, a incorporação de algumas ferramentas do PlanificaSUS e aos aprendizados advindos da pandemia. Como um elemento dificultador, observa-se a carência da previsão orçamentária de despesas inerentes a execução do PlanificaSUS e o baixo envolvimento de outros setores da SES; conforme representados no Quadro 4.

**Quadro 4:** Dimensão técnico operacional.

Elementos facilitadores	
Envolvimento dos profissionais do serviço	Valorização da planificação capilarizada entre os profissionais envolvidos
Incorporação de ferramentas do PlanificaSUS	Ferramentas de planejamento incorporadas nos planos locais
	Incorporação nos processos de trabalho em APS
	Utilização dos instrumentos da planificação nas atividades assistenciais da APS
Elementos dificultadores	
Insuficiência de previsão orçamentária para todas as necessidades	Custos não previstos para o desenvolvimento do PlanificaSUS prejudicaram a participação de vários municípios
	Carência de alternativas operacionais para adequação no projeto
Pouca flexibilidade do modelo do PlanificaSUS	Estratégias de implementar o PlanificaSUS: pouco aberto a adaptações locorregionais
	Rigidez no controle de tarefas
Menor envolvimento de outras áreas da gestão na operação do projeto	Pouco envolvimento de outros setores estratégicos da SES, além da atenção básica

APS: atenção primária à saúde; SUS: Sistema Único de Saúde.

Como mencionado anteriormente, nos municípios de pequeno porte, a gestão valorizou a formação propiciada pelo PlanificaSUS, apontando sua importância como estratégia para a reorganização dos serviços da APS. Nos municípios de maior porte houve a adesão da equipe central e, principalmente, da equipe da unidade laboratório (UL), que apresentou transformações na dinâmica de trabalho, além de assumir o papel de multiplicar, para além da sua unidade, todas as ferramentas implementadas e adotadas na articulação da APS com a AAE.

Nas unidades que participaram do PlanificaSUS, algumas ferramentas da PAS foram incorporadas ao cotidiano dos serviços, tais como: *dashboard* e PDSA, apoiados pelo plano de ação. Observou-se a valorização de uma cultura de planejamento e monitoramento, com alguns planos locais avançando na reorganização da APS, como mutirões de cadastramento, agendamento escalonado em blocos de horas e classificação de risco familiar. Outros estudos também identificaram que a PAS teria contribuído para melhorias de processos na APS, entre estes: cadastros dos usuários e registros no e-SUS, melhor organização dos fluxos dos usuários e profissionais na UBS e maior integração dos profissionais^12^.

O PlanificaSUS disponibilizou às equipes ferramentas tradicionais da gestão que, contudo, não eram utilizadas cotidianamente nos serviços. A incorporação dessas práticas contribui para organizar e qualificar a APS, bem como e melhorar o cuidado oferecido à população. Mais que uma mudança na lógica da gestão, o PlanificaSUS agregou tecnologias úteis que ampliaram a caixa de ferramentas das equipes. Alguns processos se destacaram, como: (1) o cadastro populacional, (2) a estratificação de risco, (3) organização de fluxos, (4) o agendamento de horários e (5) a digitalização. Alguns autores confirmam que mudanças na estrutura e, principalmente, nos processos deverão ser feitas na APS para a construção social do modelo da coordenação do cuidado^19^.

Na perspectiva de sistemas de atenção à saúde estruturados em RAS, é fundamental a presença equilibrada de médicos generalistas e especialistas, atuando conjuntamente em benefício das pessoas usuárias^20^.

Portanto, na perspectiva técnico-operacional, se fortalece a articulação entre a APS e a AAE quando se constroem consensos entre seus profissionais, novas ferramentas de gestão são incorporadas aos serviços e se produzem novas curvas de aprendizagem. Evangelista et al.^12^ ratificaram, em seu estudo, que processo de educação permanente ampliou o campo de atuação dos profissionais e qualificou ações de ambos os níveis de atenção, bem como consolidou a atenção compartilhada, com possibilidade do ajustamento mútuo e apoio técnico entre ambas as equipes.

Os custos operacionais e de infraestrutura para realização de atividades do PlanificaSUS não foram previstos, o que representou tensão e elemento dificultador para participação de vários municípios.

Ademais, a troca constante de tutores e a falta de acesso à rede informatizada também foram fatores que fragilizam a preparação e potência dos encontros, levando a um menor envolvimento dos participantes, com consequente repercussão no processo de ensino-aprendizagem.

Outro elemento dificultador que emergiu entre os atores que participaram da pesquisa está relacionado ao modelo adotado pelo PlanificaSUS para implantação da PAS, considerando a execução das atividades em etapas (preparatória, operacionais e controle) previstas em cronograma. Trata-se de uma proposta bastante estruturada, com poucas aberturas para flexibilização e adaptação a distintas realidades locais. E, ainda, houve relatos de excesso e rigidez de tarefas, não respeitando a dinâmica dos serviços, além do tempo limitado para realização delas.

### Dimensão Contexto

Com relação à dimensão contexto, os fatores facilitadores se relacionam à expertise das equipes estaduais, tanto na PAS quanto na implementação das linhas de cuidado analisadas. Destaca-se, ainda, a organização e articulação dos gestores municipais na constituição regional, identificada em alguns territórios. O Quadro 5 traz os principais elementos facilitadores e dificultadores desta dimensão.

**Quadro 5:** Dimensão contexto.

Elementos facilitadores	
Experiência acumulada	Existência de algumas iniciativas de articulação entre a APS e a AAE
	Linhas de cuidado existentes e em implementação nas regiões, facilitando a adesão
	Experiência prévia das SES com planificação
Organização e articulação dos gestores municipais	Articulação prévia dos municípios da região
	Compartilhamento prévio de serviços e financiamento
Elementos dificultadores	
Gestão do trabalho do SUS (SES e SMS)	Frequentes perdas de trabalhadores de saúde nas equipes assistenciais (reforma administrativa; contratos de trabalho precários; extinção do Programa Mais Médicos; pandemia covid-19)
	A pandemia modificou completamente os processos de trabalho das unidades de laboratório e o nível secundário
Relação entre SMS, SES e MS	Municípios identificaram apoio insuficiente da SES à implementação das linhas de cuidado
	Mudanças na política nacional da AB impactam no PlanificaSUS
Pandemia	Aumento do volume de trabalho da APS e fechamento da AAE em alguns períodos
	Afastamento constante de profissionais

APS: atenção primária à saúde; AAE: atenção ambulatorial especializada; SUS: Sistema Único de Saúde; AB: atenção básica; SES: Secretária Estadual de Saúde; SMS: Secretária Municipal de Saúde; MS: Ministério da Saúde.

As variáveis de contexto interno e externo tiveram forte influência nas ações de nível local e regional, para o ator capacitado na planificação, quanto ao desempenho nas ações em nível locorregional. Entretanto, quando se detectam muitos afastamentos de profissionais dos serviços ou da gestão por doenças, como notado durante a pandemia, ou por falta de coordenação regional, há prejuízos imensos na articulação APS/AAE.

Ainda assim, essas variáveis se apresentaram diversificadas e específicas, tendo em vista a construção social de cada uma das regiões. A premissa do PlanificaSUS era que elas dispusessem de um serviço ambulatorial especializado na linha de cuidado definida para cada região. Esse tipo de equipamento permitiria a concretude da integralidade na região de saúde. Foi identificado, desde a primeira visita, que existia um histórico de implantação desses serviços nos municípios sede/polos, envolvendo os distintos municípios da região.

Em contraponto aos fatores facilitadores, encontramos, também, elementos dificultadores no que se refere à dimensão contexto relacionada à gestão do trabalho no SUS, seja no âmbito das SMS, seja da SES. Foram relatados problemas de distintas ordens, que impactaram diretamente na perda da força de trabalho do SUS municipal e estadual, tais como: reforma administrativa, contratos de trabalho precários, extinção do Programa Mais Médicos e pandemia de covid-19.

Por fim, destacamos algumas dissonâncias entre as gestões dos entes federados, identificadas pelos gestores locorregionais, que tiveram impacto na implementação da PAS. Gestores municipais mostraram apreensão acerca do efetivo apoio da SES nesse processo, ao relatarem experiências nas quais o tal apoio institucional vinha sendo insuficiente na construção das linhas de cuidado. Outra tensão detectada está relacionada com os impactos das mudanças implementadas pelo Ministério da Saúde na Política Nacional da Atenção Básica e no Mais Médicos. Estas mudanças gerariam perdas de recursos para os municípios, além da necessidade de revisão de suas prioridades, o que repercutiu diretamente na continuidade do processo de implantação da PAS tal qual programado pelo PlanificaSUS.

A Figura sintetiza os resultados discutidos e aponta a importância da continuidade do processo, considerando que a pandemia redirecionou as decisões e operações do sistema de saúde para o cuidado às condições agudas, o que deixa as condições crônicas com ainda maior necessidade de articulação, para a garantia de acesso tanto na APS quanto na AAE, com integralidade e em RAS^44^.

**Figura:** Síntese agregada dos resultados pelas três dimensões analíticas.

APS: atenção primária à saúde; AAE: atenção ambulatorial especializada.

Soma-se às demandas do covid-19, as pessoas portadoras de condições crônicas, agudizadas ou não, e as condições agudas não covid-19, trazendo importantes desafios para os gestores do SUS.

## CONCLUSÕES

A avaliação da implementação foi realizada em quatro regiões de saúde pré-selecionadas, baseada na iniciativa do projeto PlanificaSUS para implantação da PAS.

Estas regiões tiveram o potencial de permitir descortinar características do processo de atenção à saúde para a implantação de novas práticas e a identificação das variáveis de contexto que influenciam as atuações técnico-políticas na saúde.

Os resultados indicaram importantes facilitadores e dificultadores para a implementação das RAS, iniciada pela qualificação da APS e da AAE nas quatro regiões estudadas.

Foi notório que muitas regiões utilizaram o projeto PlanificaSUS como uma oportunidade de articulação entre estados e municípios e uma aposta política importante para a construção de redes e linhas de cuidado, mas ainda há muitos caminhos a percorrer. Cabe considerar o papel do gestor estadual na aposta relacionada ao projeto, tanto no nível central quanto no nível regional, e o efeito de processos de mobilização da APS e amplificação de sua potência, em particular em municípios de pequeno porte.

Nesse sentido, é importante apontar que foi estabelecida uma cultura de planejamento local na APS, mesmo em contextos desfavoráveis, mas muitas vezes restrito apenas às UL. Ainda se está distante de um planejamento regional articulado, com a atenção especializada, que vá mais além da distribuição de vagas pelos distintos municípios.

Com base no referencial da construção social da APS^13^, é possível fazer uma análise a partir dos seus macro e micro processos, desenvolvidos a partir da PAS. Em outras palavras, o SUS, um sistema alicerçado e organizado a partir da APS e que se encontra em permanente busca pela melhoria do cuidado, continua sendo pauta permanente para a PAS. O projeto contribuiu para que a APS enfrente, rotineiramente, a sua capacidade de garantir a continuidade da atenção, entre seus diversos serviços.

Como possível resultado desse processo, busca-se a melhoria da estrutura para os cuidados na APS e a implantação de todos os macroprocessos estabelecidos no modelo de intervenção da PAS.
